# Assessment of fracture stability following modified minimally invasive reduction osteosynthesis system (MIROS) fixation for Neer 2 and 3-Part proximal humeral fractures

**DOI:** 10.1186/s12891-025-08600-4

**Published:** 2025-04-21

**Authors:** Osam Mohamed Metwally, Ahmed Hatem Farhan, Mahmoud Abdo Mahmoud, Hossam Fathi Mahmoud, Fahmy Samir Fahmy

**Affiliations:** Department of Orthopedic Surgery, Faculty of Medicine, Zagazig University, Sharkia, Egypt

**Keywords:** Modified MIROS, Conservative, Proximal humeral fractures, Fracture stability

## Abstract

**Background:**

Conservative management for elderly proximal humeral fractures is an acceptable option, but the fracture stability is dubious. The purpose of this study was to investigate fracture stability and functional outcomes after modified minimally invasive reduction osteosynthesis system (MIROS) and non-surgical treatment for Neer two and three-part proximal humeral fractures in elderly patients.

**Methods:**

Elderly Patients with two and three-part proximal humeral fractures who underwent modified MIROS fixation and non-operative management were retrospectively included. The Constant-Murley score, and the range of shoulder forward elevation were measured for functional assessment, while the radiological humeral head height (HHH) and humeral shaft angle (HSA) were used to verify the fracture stability. The variables in both treatment groups were compared using a two-tailed t test for independent means, with a *p* value of less than 0.05 denoting a significant difference.

**Results:**

Forty-two patients were consecutively included, with a mean follow-up of 24.05 ± 3.9 months for the modified MIROS group and 24.67 ± 4.5 months for the non-operative group. The modified MIROS group had statistically significant improvements in the Constant score and shoulder forward flexion (*p* = 0.0001), with a lower complication rate (14.3% vs. 52.3%). Moreover, the average changes in the radiological HSA and HHH were lower in the modified MIROS group at the one-year follow-up (*p* = 0.00001).

**Conclusions:**

Modified MIROS is recommended as an alternative to conservative treatment for Neer 2 and 3-part proximal humeral fractures in elderly, medically unfit patients. It is a minimally invasive procedure that provides adequate fracture stability and permits early shoulder motion, with satisfactory functional and radiologic outcomes and fewer complications.

**Level of evidence:**

Retrospective comparative cohort; level of evidence (III).

## Introduction

Proximal humeral fractures are the third most common osteoporotic fracture following fractures involving the wrist and hip in the elderly population [1]. The prevalence is higher in females, accounting for 5% of all whole-body fractures and 25% of all humeral fractures [2]. It has a bimodal distribution among the population [3]. The main mechanism of injury in young patients is high-energy trauma, such as motor accidents. In elderly patients, it follows low-energy mechanisms, such as minor home falls [4].

Neer’s [5] classification is a widely accepted scheme that categorizes proximal humeral fractures into minimally displaced, two-part, three-part, four-part, and fracture dislocation according to the angulation (> 45°) and the amount of displacement of bone fragments (> 1 cm). The choice between treatment modalities is dependent on the patient’s age, type of fracture, comminution, bone quality, and associated comorbidities [6]. The non-operative treatment is the cornerstone method for elderly proximal humeral fractures, particularly in non-displaced patterns or patients who have medical problems [7]. Several studies recorded that conservative management in fractures with widely displaced fragments sometimes has unsatisfactory functional results as it is difficult to maintain the reduction, so operative fixation is preferable for these injuries [8]. However open reduction and internal fixation (ORIF) provides angular stability for unstable fractures, the risk of infection, devascularization of bone fragments, and blood loss are the main pitfalls [9].

Reverse total shoulder is a valid option for certain geriatric complex proximal humeral fractures, but the rate of complications ranges from 12 to 68% including neurological injuries, blood loss, loosening, nonunion and periprosthetic joint infection [10].

Percutaneous K-wire fixation was described by Bohler in 1962 [11]. The main advantages were preservation of the vascular supply, no blood loss, and the capability of surgery under regional anesthesia; for these reasons, it was performed as an alternative for management of unstable fractures in the elderly with concomitant medical morbidities [12]. Many percutaneous fixation methods have been outlined in the literature, with a rate of postoperative complications of 10–21%, including loss of reduction, varus malunion, and pin tract infection [13].

The original Minimally Invasive Reduction and Osteosynthesis (MIROS) utilized multiple k-wires to correct the angular displacement and fixation of the fracture fragments. The wires in the proximal and distal segments are linked with a clamp outside the skin to provide more angular stability than traditional K-wire fixation [14]. We assumed that a modification by applying retrograde intramedullary elastic nails to fix the humeral head and replacing the clamp with Ilizarov cubes might result in more rigidity in the frame and more added stability to the fracture. The goal of the study was to compare fracture stability and clinical results following modified MIROS and conservative treatment in elderly patients with two or three-part proximal humeral fractures. It was hypothesized that the modified MIROS technique would provide better functional and radiological results with fewer morbidities.

## Patients and methods

**Fig. 1:**
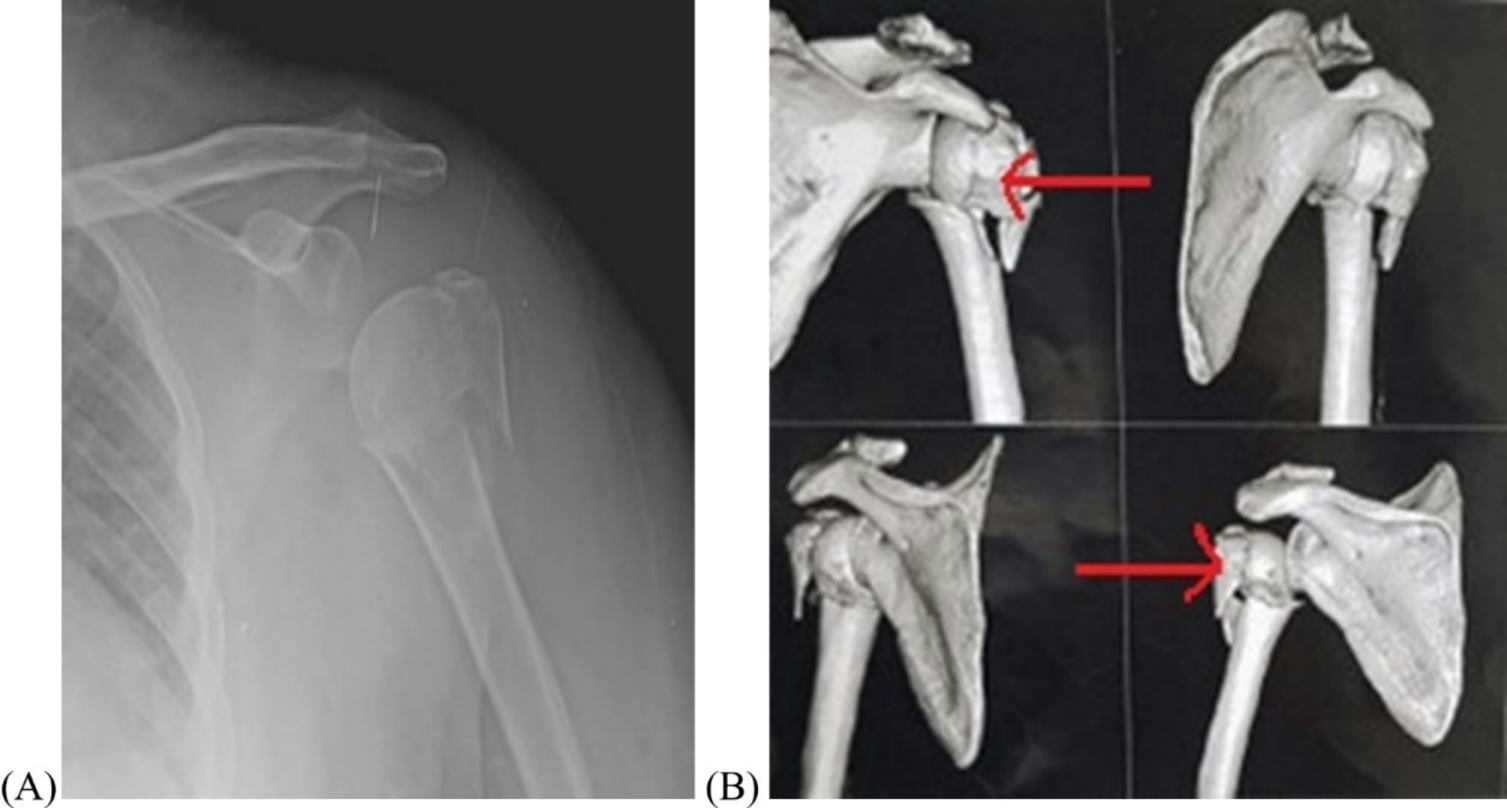
A female patient had a Neer three-part fracture on plain radiograph of the shoulder AP view (**A**) and CT scan (**B**) involving greater tuberosity and surgical neck (red arrows)

The medical files of 42 elderly patients (> 65 years) who sustained Neer two, and three-part proximal humeral fractures were retrospectively reviewed for analysis after obtaining institutional review board (IRB) authorization (ZU-IRB #101030-3-9-2023) and informed consent from the participants. The patients had surgeries between January 2020 and March 2021 at Zagazig University trauma unit. Most patients had ASA (American society of anesthesiologist) class II and III. The mean age was 67.6 ± 2.4 years for the modified MIROS group and 68.7 ± 1.3 years for the non-operative group. The criteria for exclusion were as follows: (1) Patients with ASA class I who were candidate for ORIF; (2) non-displaced fractures; (3) Neer four-part fracture; (4) fracture-dislocation; (5) open fractures; (6) fractures involving the articular surface of the humeral head; (7) concomitant glenohumeral arthritis; (8) highly comminuted calcar; and (9) patients who had a follow-up period less than 12 months or incomplete medical records. The patients were evaluated clinically regarding the mechanism of injury and their neurovascular status. Routine preoperative anteroposterior and, when possible, axillary X-ray views of the shoulder, along with a CT scan, were utilized to delineate the type and the extent of fracture [Figure 1]. Table 1 demonstrates the characteristics of the patients for each treatment group.

**Table 1:** Demographic criteria of the treatment groups

		Modified MIROS group (*N* = 21)	Non-operative group (*N* = 21)	*P* value
**Mean age (range**,** years)** **Confidence interval (95%)**		67.6 ± 2.4 (65–73) [66.5–68.6]	68.7 ± 1.3 (67–72) [68.1–69.2]	0.619
**Sex**	Male	6 (28.6%)	8 (38.1%)	0.744
	Female	15 (71.4%)	13 (61.9%)	
**Side**	Dominant	14 (66.7%)	11 (52.4%)	0.530
	Nondominant	7 (33.3%)	10 (47.6%)	
**Mechanism of injury**				0.696
Road traffic accidents		3 (14.3%)	5 (23.8%)	
Falls		18 (85.7%)	16 (76.2%)	
**Neer’s classification**				0.606
Three-parts		20 (95.2%)	18 (85.7%)	
Two-parts		1 (4.8%)	3 (14.4%)	
**ASA score**				0.380
**II**		6	4	
**III**		11	9	
**IV**		4	8	

Values are illustrated as numbers (n), percentages (%), and means ± SD_s_ (range). ASA; American Society of Anesthesiologists for physical status

### Surgical technique

**Fig. 2:**
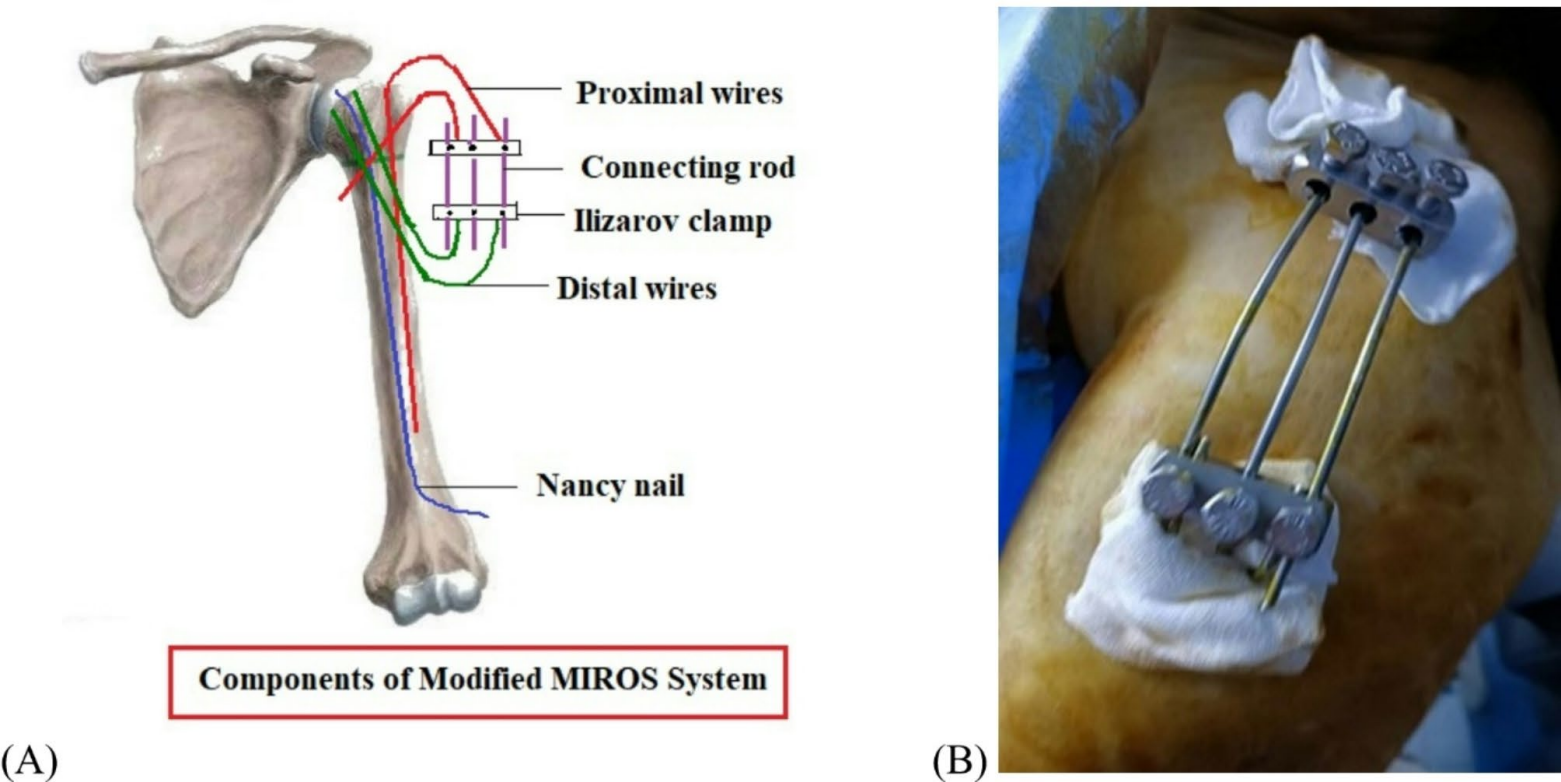
An intraoperative picture and schematic diagram show the components of the modified MIROS frame during surgery (**A**, **B**)

An intravenous injection of 1 g of cefazolin was given 30 min before the beginning of surgery. All procedures were done under regional anesthesia by a single surgeon who had more than ten years of surgical experience in external fixation. The patients were placed supine in a 30-degree semi sitting position, keeping the affected shoulder off the edge of the operating table to facilitate imaging. The fracture was manipulated under the direction of a c-arm intensifier to check the quality of reduction. First, two or three elastic Nancy nails (3 mm in diameter) were inserted in a retrograde fashion through the medullary canal of the distal segment making a small entry hole 5 cm above the lateral epicondyle using a small awl to hold the humeral head in a reduced position. The initial proximal K-wire was inserted through greater tuberosity, engaging the medial cortex to secure the greater tuberosity. A second K-wire of 2 mm in diameter passed from the largest portion of the humeral head down to the distal fragment. Another two K-wires were directed cranially from the lateral cortex of the distal fragment to the head of the humerus to fix the fracture. The proximal and distal wires were bent over and interconnected with two Ilizarov cubes and three connection rods [Figure 2]. The skin was stitched over the buried distal end of the elastic nail.

### Postoperative care and follow-up

**Fig. 3:**
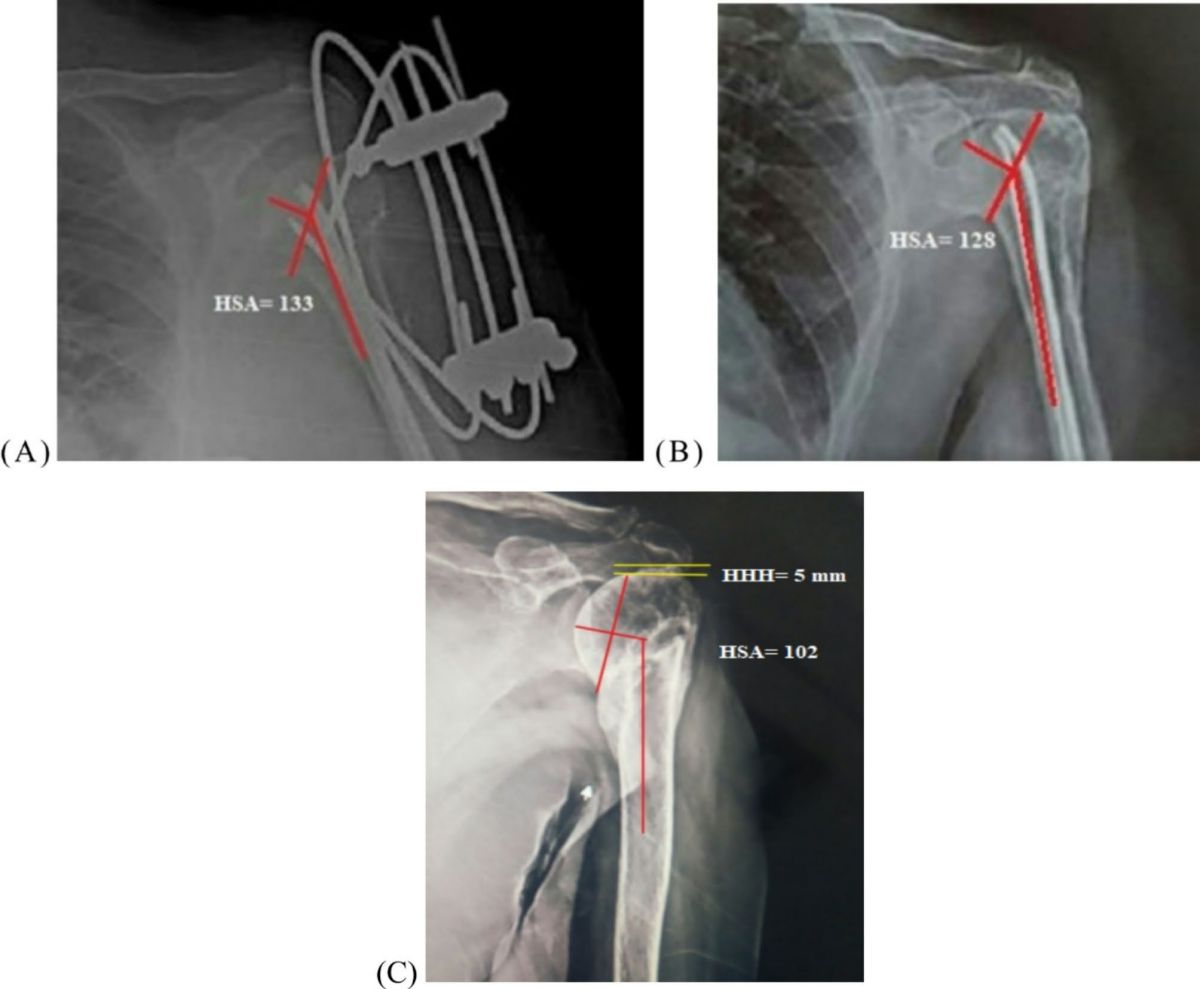
The HSA angle in the initial radiograph is 133° (**A**) and 128° at 12 months postoperative (**B**) after modified MIROS fixation. The HHH was − 5 mm, and the HSA was 102° at one-year radiograph following conservative treatment, indicating varus malunion (**C**)

A prophylactic antibiotic was continued for 24 h postoperatively. The arm was held in a sling for 6 weeks, with the removal of the sutures after 2 weeks for the modified MIROS group. Passive motions of the shoulder commenced as much as possible for the modified MIROS group and after 4 weeks for the group treated conservatively. The wires are removed after the fracture union. The Constant-Murley score [15] and the range of shoulder forward flexion were documented at the last follow-up for clinical evaluation. Plain radiographs of the shoulder were obtained one day after surgery and then every month until the time of union. The union is defined as a painless fracture site with a radiographic bridging callus. The head shaft angle (HSA) and humeral head height (HHH) were recorded on the AP view at 12-month postoperative radiographs for assessment of fracture stability using ImageJ software by a radiologist blinded to treatment [Figure 3]. The HSA was defined as the intersection between a line parallel to the center of the humeral shaft and a line perpendicular to a line connecting the superior and inferior articular margins. The distance between two lines tangent to the tip of the greater tuberosity and the highest point of the articular margin was the HHH. Loss of reduction was considered as a HSA < 120°, with a change of > 10° [16] and a HHH difference of > 3 mm [17].

### Statistical analysis

The normal distribution of the data was assessed using the Shapiro-Wilk test. The quantitative values were expressed as means and standard deviation, with the calculation of a 95% confidence interval for means. The continuous variables of the treatment groups were compared using the independent t test and Mann-Whitney test, while the difference between ordinal data was evaluated using Fisher’s exact test. The minimal clinically important difference (MCID) for the Constant score was estimated as one and half the SD of the control group to assess the clinical significance. The HHH and HSA were correlated with the Constant score using Pearson’s coefficient correlation test. Statistical significance was indicated for a P value < 0.05 for all tests. The post hoc analysis of the test power was 93.9% according to the given sample size, effect size of the HSA between groups, and 0.05 alpha error. The Statistical Package for Social Sciences (SPSS) (IBM, version 25.0, Inc., Chicago, IL) was used for the statistical processing of data.

## Results

A total of 47 patients with Neer two and three-part proximal humeral fractures were referred to our trauma unit. Forty-two patients were selected for inclusion in this study after eliminating two cases with incomplete data records and three patients underwent ORIF. Twenty-one patients were operated on using the modified MIROS technique, while the others were treated non-operatively in the outpatient clinic using an arm sling. There was no statistical difference between treatment groups regarding age, sex, side of dominance, mechanism of injury, classification of fracture, or health status [Table 1].

The mean follow-up time was 24.05 ± 3.9 (range, 13–32 months) for the modified MIROS group and 24.67 ± 4.5 (range, 12–34 months) for the non-surgical group (*p* = 0.63). The mean time before surgery in the modified MIROS group was 3.5 ± 1.7 days. The modified MIROS group reported a better Constant score than the conservative group (83.6 ± 3.4 vs. 75.1 ± 6.7), which was statistically significant (*p* < 0.0001). There was no clinically significant difference in the Constant score between treatment groups as the estimated MCID was 10.05, which is higher than the actual difference between means (8.5). Also, the mean range of shoulder forward elevation showed a significant difference between both groups (*p* < 0.00001) [Table 2]. No cases of nonunion were recorded in either group, but the modified MIROS group had a shorter union time than the conservative group (10.48 ± 1.5 vs. 12.9 ± 2.1 weeks) (*p* < 0.0001).

The follow-up means for HSA and HHH reveal significant statistical difference between the treatment groups in the 1-year postoperative radiographs (*p* = 0.00001) [Table 2]. The average change in the HSA was 2.7° for the modified MIROS group and 15.2° for the non-operative group (*p* = 0.00001). Additionally, there was a statistically significant change in the HHH between both groups (*p* = 0.00001) [Table 2]. We found that the Constant score had a statistically significant positive correlation with the HSA (*r* = 0.93 and *p* < 0.00001) and the HHH (*r* = 0.97 and *p* < 0.00001) on 12-month postoperative radiographs in the conservative group.

The rate of complications revealed a significant difference between treatment groups (*p* = 0.006). The modified MIROS group reported one case of varus malposition, and two cases of pin tract infections that were resolved after removal of the frame. The non-operative group recorded nine cases of varus malunion, and two stiff shoulders with global restriction of the ROM.

**Table 2:** Final clinical and radiological results for the treatment groups

	Modified MIROS group	Non-operative group	95% CI of the difference	*P* value
**Final mean range of forward elevation (95% CI) (degree)**	155.9 ± 5.19 (153.5–158.2)	122.28 ± 6.3 (119.4–125.1)	(30.02–37.2)	0.00001^*^
**Final mean Constant score (95% CI)**	83.6 ± 3.4 (82.05–85.1)	75.1 ± 6.7 (72.05–78.15)	(5.2–11.8)	0.0001^*^
**Mean time of union (95% CI) (weeks)**	10.48 ± 1.5 (9.8–11.2)	12.9 ± 2.1 (11.9–13.8)	(1.3–3.5)	0.0001*
**Mean HSA (degree)**				
Initial follow-up	124.4 ± 3.9	118.9 ± 2.6	-	-
12-months follow-up	121.9 ± 3.3	103.7 ± 4.7		0.00001^*^
Mean change (95%CI)	2.7 ± 1.6 (1.9–3.4)	15.2 ± 4.4 (13.1–17.2)		0.00001^*^
**Mean HHH (mm)**				
Initial follow-up	6.1 ± 2.6	3.6 ± 2.1	-	-
12-months follow-up	5.4 ± 2.6	0.1 ± 2.2		0.00001^*^
Mean change (95%CI)	0.67 ± 0.5 (0.4–0.8)	3.5 ± 0.6 (3.2–3.7)		0.00001^*^
**Complications**	3 (14.3%)	11 (52.3%)	-	0.0203 ^*^

CI: confidence interval, MIROS: Minimally Invasive Reduction Osteosynthesis System, HSA: Humeral shaft angle. HHH: Humeral head height. ^*^P < 0.05 indicates a significant difference

## Discussion

This study identified that the modified MIROS group had superior fracture stability compared to the non-surgical group, as measured by the radiological results of the HHH and the HSA. Furthermore, the modified MIROS group had statistically significant improvements in functional outcomes in terms of the Constant score and range of shoulder forward flexion, as well as a decreased rate of complications. These findings endorse the study hypothesis. To our current knowledge, this is the first to evaluate fracture stability after modified MIROS fixation in elderly patients with unstable proximal humeral fractures.

The displaced patterns constitute 13–16% of all proximal humeral fractures, and most of them necessitate surgical intervention [18]. The rate of operative interventions has increased in the past few years to avoid the detrimental and suboptimal functional results of conservative therapy for these fractures [19].

Splitting of the humeral head, fracture of the anatomical neck, comminuted irreparable tuberosities, associated shoulder osteoarthritis, and failure of other treatment modalities are candidate for reverse total shoulder replacement [20]. A recent systematic review concluded that reverse shoulder arthroplasties had the best functional outcomes compared to the other treatment modalities, however, the range of surgical complications varies widely in literature according to the age of patients, health condition, and indications, with more hazards among patients with significant medical illness [10].

Fixation of fractures involving three or four bone fragments is problematic because of the limited bone stock in elderly patients [21]. Various fixation techniques have been utilized in literature; however, there is no evidence of the superiority of a single technique [22]. Regardless of the method used, the aim of surgery is to provide efficient fracture stability with preservation of bone vascularity [23]. Percutaneous fixation is best suited for two-part, three-part with minimal comminution of the calcar, and valgus impacted four-part fractures [20]. It is the simplest technique used in older patients with medical comorbidities; however, varus malunion and pin migration are the main drawbacks [13]. MIROS fixation was previously developed to increase the stiffness of fixation and minimize the loss of reduction by transferring the stresses from the cancellous bone to the more rigid outer cortex [12], but fracture stability was not fully studied with this technique. Many authors asserted that MIROS fixation is a reasonable alternative to conservative therapy that can be performed under regional anesthesia, particularly in older age groups with medical illnesses [6, 12]. We found associated medical diseases in 71.4% of the modified MIROS group and 80.9% of the conservative group.

Consistent with previous publications [4, 12], this study confirmed that these fractures were more likely in females (71.4% in the modified MIROS group and 61.9% in the non-operative group), with low-energy collisions serving as the primary mechanism of injury in both treatment groups.

When compared to conservative management, our study demonstrated a striking statistical improvement in the Constant score after the modified MIROS method. The modified MIROS group had an average score of 83.6, whereas the non-operative group had a score of 75.1. This corresponded to that described by D’Ambrosi et al. [24], who reported an average Constant score of 88.9 in 32 patients treated with an external fixation device. In addition, Maluta et al. [6] treated 18 elderly patients using external fixation with an equivalent Constant score (85 ± 9.8). The lower Constant score of 60 points observed by Carbone et al. [12] in 31 patients treated with the MIROS technique may be attributed to the higher mean age of 80.7 ± 7 years and the presence of two cases with postoperative avascular necrosis. Carbone et al. [12] and Bhavsar et al. [25] discovered statistically significant increments in the Constant score following percutaneous external fixation of unstable proximal humeral fractures with respect to the K-wire technique (89.18 vs. 78.64). Because of the risks pertaining to ORIF, Bai et al. [26] and Cui et al. [27] found that their patients exhibited lower mean Constant scores of 76.7 ± 10.1 and 73 ± 9.94, respectively. A meta-analysis by Oldrini et al. [28] displayed no significant difference in the Constant score between ORIF and minimally invasive plate osteosynthesis (MIPO) among different types of proximal humeral fractures. A recent systematic review by Soler-Peiro et al. [29] displayed a lower constant score of 64.5 following non-surgical therapy for elderly 3-parts proximal humeral fractures.

One of the valuable advantages of MIROS fixation over percutaneous pinning is that it permits an early passive range of shoulder mobility [12]. According to our findings, patients in the modified MIROS group initiated passive motion earlier than those in the conservative group. The patients in the modified MIROS group achieved a final mean shoulder elevation of 155.9 ± 5.19° and 122.28 ± 6.3° in the non-operative group, which was statistically relevant. The same findings were documented by Carbone et al. [12], Bhavsar et al. [25], and Roberson et al. [30]. Cui et al. [27] noticed that patients treated with proximal humerus internal locked osteosynthesis system (PHILOS) plating had a lower mean shoulder abduction of 140.64 ± 20.34° owing to soft tissue dissection during the surgical approach.

Neer type 3 and type 4 fractures, in some instances, need stable fixation methods. Loss of reduction occurs frequently with percutaneous pinning [31]. Carbone et al. [12] stated that the MIROS technique should replace traditional K-wire fixation because it offers higher fracture stability. We modified the classic MIROS construct by inserting two or three elastic intramedullary nails and substituting the original clamp with Ilizarov cubes. We suggest that the elastic nails keep the humeral head reduced and in proper alignment with the shaft. The MIROS approach has not previously been studied for loss of reduction. Varus malunion has a detrimental effect on shoulder function, as it puts the supraspinatus muscle at a mechanical disadvantage that requires a higher force to elevate the arm [32]. Many studies have demonstrated that the change in the radiological HSA is considered a predictor for loss of fracture stability [26, 33]. Bai et al. [26] and Greiner et al. [34] showed that a HSA < 120° and a change of more than 10° are the cut-off levels of varus malalignment. Another prognostic factor for fracture stability is the HHH, with changes of more than 3 mm having a negative influence on shoulder abduction [17, 26]. We reported an average HSA of 124.4° in the modified MIROS group and 118.9° in the conservative group upon the initial postoperative radiographs; however, the change in the HSA was greater in the non-operative group at the 1-year follow-up (2.7° vs. 15.2°). Furthermore, the average change in the HHH was higher in the conservative group at 12 months postoperatively (0.67 mm vs. 3.5 mm). The results of the radiological parameters in the modified MIROS group were comparable to those obtained by Bai et al. (Δ HSA = 3.7° and Δ HHH = 1.7 mm) [26] and Cui et al. (Δ HSA = 3.1° and Δ HHH = 1.1 mm) [27] using the ORIF technique with locked plates. Moreover, we noticed a strong positive correlation between the HSA and HHH and the functional Constant score, as previously had been confirmed by Bai et al. [26].

No cases of nonunion were discovered in either group, but the modified MIROS group had a shorter average time of union (10.48 vs. 12.9 weeks). Our results matched those recorded by Monga et al. [35], who treated 20 patients with unstable proximal humeral fractures using an AO external fixator. Gupta et al. [14] recorded a shorter duration of union of 6.5 weeks, which could be explicated by the smaller number of enrolled patients (16 Neer type 2 and type 3 fractures).

The overall complication rate in the modified MIROS group was lower than that in the non-surgical group (14.3% vs. 52.3%). Our results were close to those stated by Carbone et al. [12], who reported a total complication rate of 10.7% in the MIROS group. Furthermore, Maluta et al. [6] had a similar complication rate of 11.76%. We had only one case of varus deformity in the modified MIROS group and nine cases in the non-operative group (*p* = 0.008), like Carbone et al. [12], who noticed one patient with moderate displacement in the MIROS group. Bhavsar et al. [25] did not show varus collapse in the external fixator group. Soler-Peiro et al. [29] recorded a 21% of varus malposition in 133 patients treated conservatively.

This study had some limitations. First, it should be carried out on a larger scale of patients and performed using single surgeon for generalizability of the results; however, the post hoc analysis revealed that the number of recruited patients was sufficient to produce good statistical power. Second, the study was retrospective, and extra prospective studies are advisable to minimize the selection bias. In addition, delayed complications, such as glenohumeral arthritis, need a longer duration of follow-up. Also, there was a disparity between the statistical and clinical significance for the functional score due to the small sample size. This study lacks comparison with other treatment modalities like percutaneous pinning and MIPO fixation. Finally, the radiological measurements of the HSA and HHH could be skewed by arm malposition during imaging; therefore, further prospective estimation is recommended.

## Conclusions

We concluded that the modified MIROS produced satisfactory improvements in the radiological HHH and HSA for Neer 2 and 3-part proximal humeral fractures in elderly patients. It has better clinical outcomes and fracture stability compared to conservative treatment. It is minimally invasive, so we advocate it as a good substitution to non-operative management for elderly proximal humeral fractures with poor general condition. Additional research comparing the modified MIROS fixation with other treatment options will be demanded in the future.

## Data Availability

There is no research data outside the submitted manuscript file.

## Abbreviations

MIROS: Minimally invasive reduction osteosynthesis system
ORIF: Open reduction and internal fixation
HSA: Head shaft angle
mm: Millimeter
SD: Standard deviation
SPSS: Statistical Package for Social Sciences
g: Gram
HHH: Humeral head height
CI: Confidence interval
